# Dopaminergic tone regulates transient potassium current maximal conductance through a translational mechanism requiring D1Rs, cAMP/PKA, Erk and mTOR

**DOI:** 10.1186/1471-2202-14-143

**Published:** 2013-11-13

**Authors:** Edmund W Rodgers, Wulf-Dieter Krenz, Xiaoyue Jiang, Lingjun Li, Deborah J Baro

**Affiliations:** 1 Department of Biology, Georgia State University, Atlanta 30303, Georgia; 2 Neuroscience Institute, Georgia State University, Atlanta 30303, Georgia; 3 School of Pharmacy and Department of Chemistry, University of Wisconsin, Madison, WI 53705-2222, USA

## Abstract

**Background:**

Dopamine (DA) can produce divergent effects at different time scales. DA has opposing immediate and long-term effects on the transient potassium current (I_A_) within neurons of the pyloric network, in the *Panulirus interruptus* stomatogastric ganglion. The lateral pyloric neuron (LP) expresses type 1 DA receptors (D1Rs). A 10 min application of 5-100 μM DA decreases LP I_A_ by producing a decrease in I_A_ maximal conductance (G_max_) and a depolarizing shift in I_A_ voltage dependence through a cAMP-Protein kinase A (PKA) dependent mechanism. Alternatively, a 1 hr application of DA (≥5 nM) generates a persistent (measured 4 hr after DA washout) increase in I_A_ G_max_ in the same neuron, through a mechanistic target of rapamycin (mTOR) dependent translational mechanism. We examined the dose, time and protein dependencies of the persistent DA effect.

**Results:**

We found that disrupting normal modulatory tone decreased LP I_A_. Addition of 500 pM-5 nM DA to the saline for 1 hr prevented this decrease, and in the case of a 5 nM DA application, the effect was sustained for >4 hrs after DA removal. To determine if increased cAMP mediated the persistent effect of 5nM DA, we applied the cAMP analog, 8-bromo-cAMP alone or with rapamycin for 1 hr, followed by wash and TEVC. 8-bromo-cAMP induced an increase in I_A_ G_max_, which was blocked by rapamycin. Next we tested the roles of PKA and guanine exchange factor protein activated by cAMP (ePACs) in the DA-induced persistent change in I_A_ using the PKA specific antagonist Rp-cAMP and the ePAC specific agonist 8-pCPT-2′-O-Me-cAMP. The PKA antagonist blocked the DA induced increases in LP I_A_ G_max_, whereas the ePAC agonist did not induce an increase in LP I_A_ G_max_. Finally we tested whether extracellular signal regulated kinase (Erk) activity was necessary for the persistent effect by co-application of Erk antagonists PD98059 or U0126 with DA. Erk antagonism blocked the DA induced persistent increase in LP I_A_.

**Conclusions:**

These data suggest that dopaminergic tone regulates ion channel density in a concentration and time dependent manner. The D1R- PKA axis, along with Erk and mTOR are necessary for the persistent increase in LP I_A_ induced by high affinity D1Rs.

## Background

**Figure 1:**
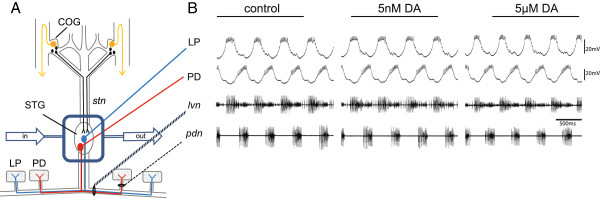
**Stomatogastric nervous system and experimental method. A**. The stomatogastric nervous system (STNS) was dissected from the animal and pinned in a Sylgard dish. **A** petroleum jelly well was constructed around the stomatogastric ganglion (STG). There are ~30 neurons in the STG; two are drawn. Saline, with or without drugs, was superfused into the well surrounding the STG. Neurons in the commissural ganglia (COG) and esophageal ganglion provide descending modulation that remained intact until voltage-clamp. LP was identified using a combination of intra and extracellular recordings. **B**. Pyloric neurons spontaneously produce a triphasic rhythmic output (Control-Left Traces). The top two traces represent intracellular recordings from the lateral pyloric (LP) and pyloric dilator (PD) cells, while the bottom traces represent extracellular recordings taken from the lateral ventricular nerve (lvn) and pyloric dilator nerve (pdn). The application of 5nM DA produces no change in the LP rhythmic output (Middle Traces). Application of 5 μM DA (Right Traces), however, alters LP activity [14]. In 5 μM DA cycle frequency is increased and LP burst duration is a decreased [24].

Neuromodulators can produce a multitude of different effects depending on context, timescale, and concentration. DA, for example, has actions on the scale of milliseconds, during error detection [1], to minutes and hours with its effects on volitional movement and cognition [2]. In most systems, DA transmission is both tonic and phasic [3]. Using the stomatogastric nervous system (STNS, Figure 1A) in the spiny lobster, *Panulirus interruptus*, we recently demonstrated that these two types of transmissions can act over distinct time scales to produce opposing effects on the same cell type [4].

The STNS comprises several motor networks and has long served as an ideal model system for studies of neuromodulation [5]. The pyloric circuit is a 14-neuron network located exclusively within the stomatogastric ganglion (STG, Figure 1A) that is modulated by DA [6]. The STNS dopaminergic system is well defined [7-15]. L-cells within the commissural ganglia (COGs, Figure 1A) secrete DA into the hemolymph [15]. Since the STG resides in a blood vessel and is bathed by hemolymph [5], this neurohormonal DA serves as a source of tonic DA transmission to pyloric neurons, predicted to be in the pM-nM range [5,16]. In addition, modulatory DA projection neurons in the COGs use volume transmission whereby DA is released into open synapses and diffuses to its target sites before reuptake [10]. In other systems volume transmission results in tonic nM DA in the extracellular space that can rise to μM levels near the release sites of bursting DA neurons [17-19]. DA receptors are divided into two broad classes, type 1 (D1Rs) and type 2 (D2Rs). The lobster genome contains two D1Rs and one D2R [7,8]. These receptors signal through canonical pathways in the native system [9,14] and behave exactly like their mammalian counterparts when expressed in human embryonic kidney cells [7,8].

In order to better understand the roles of tonic and phasic DA transmissions, we have examined the effects of nM vs. μM DA on identified pyloric neurons. The data suggest tonic and phasic DA have distinct roles because the two concentrations produced opposing persistent vs. immediate effects on I_A_, respectively [4]. The channels mediating I_A_ are encoded by the shal (Kv4) gene in crustaceans [20-22]. I_A_ is active at sub-threshold voltages, and helps determine the rate of post-inhibitory rebound and spike frequency in pyloric neurons [23].

There is one lateral pyloric neuron (LP) in the pyloric circuit that expresses D1Rs but not D2Rs [14]. Pyloric neurons show spontaneous, rhythmic oscillations in membrane potential and burst firing (Figure 1B). A 10 min bath application of nM DA has no immediate effect on neuronal output, but bath application of μM DA immediately alters LP activity (Figure 1B), including an increase cycle frequency, a decrease burst duration, and a phase advance mediated, in part, by decreasing LP I_A_[6,14,24]. The threshold for this action is ~ μM [14] and is therefore likely mediated by low affinity D1Rs. Whereas nM DA has no immediate effect, it can act at high affinity LP D1Rs to persistently alter LP I_A_: A 1 hr application of 5 nM DA followed by 3 hr wash produced a persistent ~25% increase in LP I_A_ G_max_ relative to controls that did not receive DA [4].

The signaling pathways that transduce DA’s immediate and persistent effects appear to be distinct. Similar to the situation in mammals [25], lobster D1Rs can couple with Gs and Gq [7,9]. The immediate decrease in LP I_A_ was mediated by a D1R-AC-cAMP-PKA dependent pathway [14]. The pathway mediating the DA-induced persistent increase in LP I_A_ is unknown, but it is both translation- and mTOR-dependent [4]. Several intracellular signaling pathways can modulate the activity of the serine-threonine kinase, mTOR [26-29]. The goal of this work was to understand the dose and time dependencies and the signaling proteins involved in the DA-induced, persistent increase in LP I_A_. Here we show that dopaminergic tone regulates I_A_ density through the D1R-PKA axis, Erk and mTOR.

## Results

### The persistent effect is both time and dose dependent

**Figure 2:**
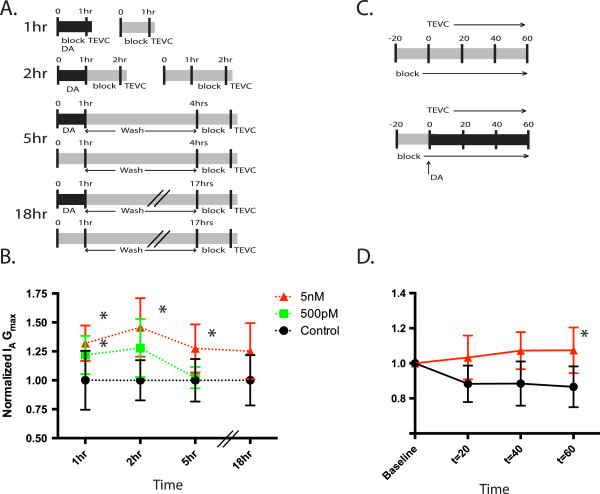
**I**_**A **_**regulation by DA is both time and dose dependent. A**. Experimental design: After identification, 1 hr, 2 hr, 5 hr and 18 hr experiments were performed as diagramed. Experiments were concluded after TEVC measurement of I_A_. **B**. Each experiment was normalized by the mean for the control group and normalized measures of LP I_A_ were plotted over time for the experiments diagramed in 2A. Data points represent normalized I_A_ G_max_ for each treatment group ± S.D. Each treatment group is independent. Asterisks indicate significant differences from control, Dunnet’s post hoc test, p < 0.05 (see text). Dashed lines indicates the within treatment trend overtime. X-axis not drawn to scale. **C**. In these experiments I_A_ was measured repeatedly throughout a 1 hr DA or saline application, as diagramed. After an initial application of blocking saline, I_A_ was measured every 20 min in the presence or absence of DA. **D**. For each individual experiment in 2C, the values were normalized to t = 0. Normalized means ± S.D. for each time point are shown. Asterisks represent significant differences between treatments determined by post-hoc analysis, p < 0.05.

We previously showed that a 1 hr application of 5 nM and 5 μM DA both produced a ~25% increase in LP I_A_ G_max_ measured 4 hr after DA washout [4]. The fact that both doses produced equivalent responses suggested that DA was acting at high affinity receptors. Here we further examined the dose dependency of the response using two DA concentrations (500 pM, 5 nM). After dissection and cell identification, a 2-5 hr process, a given concentration of DA was or was not (control) bath applied to the STG for 1 hr, and LP I_A_ was immediately measured at the end of the application, before DA washout using two-electrode voltage clamp (TEVC) (Figure 2A). Data for each time point was normalized by the mean control value. LP I_A_ G_max_ was significantly increased in 500 pM and 5 nM, relative to control preparations (ANOVA F_2,51_ = 6.728, p = 0.0026; Dunnet’s post hoc 5 nM vs ctrl, p < 0.01, 500 pM vs ctrl, p < 0.05) (Figure 2B). In another series of experiments, 50 pM DA was also applied (not shown), but was not significantly different than control, and was dropped from subsequent time points. Voltage dependencies were not altered by any concentration of DA tested (ANOVA: activation, p = 0.64, inactivation, p = 0.81).

We next examined if the effect persisted upon DA washout. Experiments were repeated for control, 500 pM and 5 nM preparations. DA or saline (control) was applied for 1 hr and then DA was washed out for 1 hr, 4 hr, or 18 hrs followed by TEVC in blocking saline (see Methods) to measure LP I_A_ (Figure 2A). Data for each experiment were normalized by the mean for control at that time point. Control means varied less than 10% between 1 hr and 18 hr. After a 1 hr DA washout (i.e. 2 hr time point), the effect of 500 pM DA on LP I_A_ G_max_ was no longer significant, whereas the significant increase produced by 5 nM DA was sustained (ANOVA F_2,15_ = 6.51, p = 0.0101, Dunnet’s post hoc, 5 nM vs ctrl, p < 0.01) (Figure 2B). After a 4 hr DA washout (i.e., 5 hr time point) average LP I_A_ G_max_ decreased to control levels in the 500 pM treated preparations but remained significantly elevated in the 5 nM treated preparations compared to control (ANOVA F_2,20_ = 5.411, p = 0.013, Dunnet’s post hoc 5 nM vs ctrl, p < 0.01, Figure 2B). I_A_ G_max_ remains elevated out to 18 hrs after DA administration [4] (Figure 2B).

The previous experiments revealed that the persistent effect of nM DA was observable, compared to controls, by 1 hr after the start of DA administration. To examine the time course for the development of the DA mediated increase in I_A_ we measured I_A_ repeatedly during a 1 hr 5 nM DA or saline (control) application (Figure 2C). To more carefully examine changes over time, we normalized all the values to t = 0 (Figure 2D) (There were no differences at t = 0 between control and DA treated preparations (t-test, p = 0.19)). We then performed mixed-model repeated measures ANOVA with time as the within-subjects factor and treatment (5 nM DA vs. Control) as the between-subjects factor. There was a significant effect of treatment (F_1,9_ = 7.10, p = 0.026), but not of time (F_2,9_ = 3.05, p = 0.0975). Post hoc comparisons, with Dunn-Sidak adjustments, revealed significant differences between treatments at 60 min (p = 0.0247) (Figure 2D). By 60 min, average I_A_ G_max_ increased by ~10%, in DA-treated preparations and decreased by ~13% in control preparations.

### The persistent effect is mediated by increased cAMP

**Figure 3:**
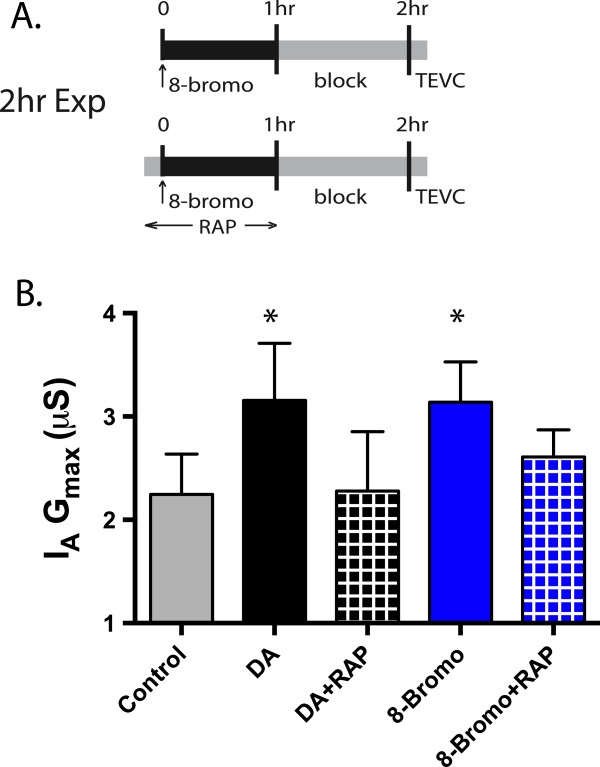
**cAMP analogue produces an increase in I**_**A **_**G**_**max**_**. A**. 50 μM 8-Bromo-cAMP (with or without rapamycin) was applied to an ongoing rhythm for 1 hr, followed by a 1 hr block and TEVC for I_A_. Rapamycin was applied 10 min prior to 8-bromo-cAMP administration. These data were compared to 2 hr control, 5 nM DA, or 5 nM DA + RAP. **B**. The data for each treatment group ± S.D were plotted. Asterisks indicate significant differences from control. Data was analyzed using ANOVA’s with Dunnett’s post hoc tests. 8-Bromo cAMP persistently increased I_A_ G_max_, while Rapamycin blocks or attenuates those effects.

Our next goal was to identify signaling molecules involved in the DA-induced, mTOR- and translation-dependent, persistent increase in LP I_A_. LP exclusively expresses D1Rs [14], of which there are 2 types that couple with Gs (D1α_Pan_) or Gs & Gq (D1β_Pan_) [7]. To first examine whether the persistent effect on LP I_A_ was mediated by cAMP, we applied the cAMP analogue, 8-bromo-cAMP or saline (control) for 1 hr followed by a 1 hr block and TEVC to measure LP I_A_ (Figure 3A). We used the lowest effective dose reported in this system [14]. Application of 8-bromo-cAMP significantly and persistently elevated LP I_A_ G_max_ by 40% compared to saline controls (t-test, p = 0.0034), while voltage dependence was not affected (t-test, p = 0.98.). Interestingly, the magnitude of the increase in LP I_A_ G_max_ produced by 8-Bromo-cAMP was very similar to that produced by 5 nM DA in the 2 hr experimental paradigm (5 nM mean ± S.E.M.: LP I_A_ G_max_ 3.16 ± 0.25, 8-Bromo-cAMP LP I_A_ G_max_ 3.14 ± 0.16, Figure 3B). To determine if the cAMP mediated persistent increase in LP I_A_ depended upon mTOR, we repeated the experiments except that the mTOR antagonist, rapamycin (100 nM), was co-applied with 8-Bromo-cAMP or 5 nM DA (Figure 3A). We then compared those groups to saline alone or saline + 5 nM DA (Figure 3B). Rapamycin reduced the 5 nM DA and 8-bromo-cAMP induced increase in LP I_A_ G_max_ (ANOVA, F_4,25_ = 6.02, p = 0.0016, Dunnet’s Post Hoc: Ctrl vs 5nM DA, p < 0.05, Ctrl vs 8-Bromo, p < 0.05, Ctrl vs 5 nM + RAP n.s., Ctrl vs 8-Bromo + RAP, n.s.) suggesting cAMP at least partially mediates the D1R-induced persistent increase in LP I_A_ G_max_.

### cAMP acts through PKA to increase I_A_ G_max_

There are several known downstream effectors of cAMP [30], notably PKA [31], ePACs [32,33], and cyclic-nucleotide gated channels [34]. We first tested whether cAMP mediated its effects on LP I_A_ through ePAC by employing the ePAC specific agonist, 8-pCPT-2′-O-Me-cAMP. This cAMP analogue has been used successfully to differentially activate ePAC1/2 as opposed to PKA [35] in a host of phylogenetically divergent animals, including crustaceans [36]. We applied 50 μM 8-cpt-cAMP or saline (control) for 1 hr, followed by a 1 hr wash and TEVC to measure LP I_A_. 8-cpt-cAMP had no effect on LP I_A_ G_max_ relative to control (t-test, p = 0.72), suggesting that the persistent effect of DA on LP I_A_ was not mediated through ePAC activation. At present there are no effective antagonists for ePACs.

**Figure 4:**
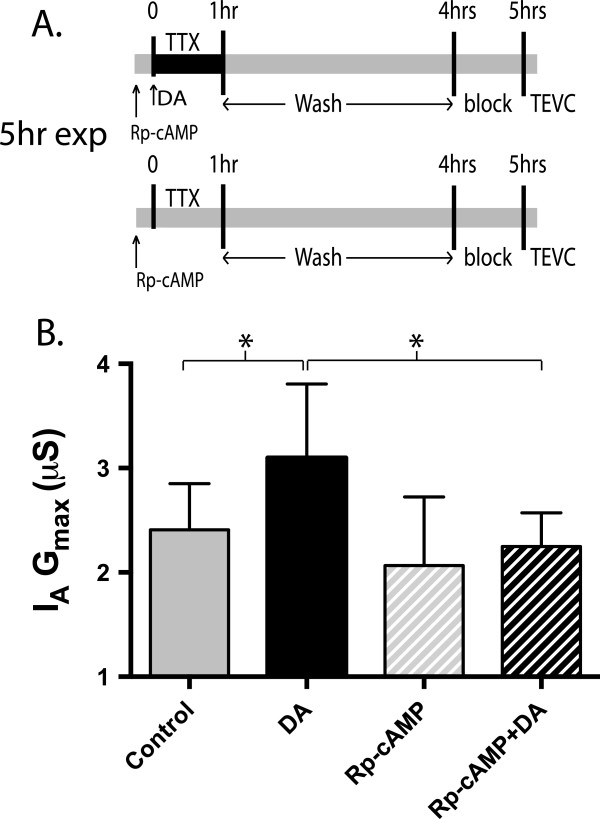
**PKA antagonist Rp-cAMP blocks DA induced increase in I**_**A **_**G**_**max**_**. A**. The specific PKA antagonist Rp-cAMP was applied in conjunction with TTX for 1 hr (with or without DA) followed by a 3 hr washout, and 1 hr block. Rp-cAMP and TTX were applied 10 min before administration of DA. **B**. 1 mM Rp-cAMP blocked the DA induced increase in I_A_ G_max,_ and was significantly different than TTX + DA alone, indicating that the persistent effect is dependent of PKA. Data were analyzed with ANOVAs, Tukey’s post hoc that makes all pairwise comparisons and plotted as mean ± S.D.

To determine if PKA mediated the D1R-induced persistent increase in LP I_A_ G_max_, we applied the specific PKA antagonist, Rp-cAMP for 1 hr with 5 nM DA and TTX, followed by 3 hr wash and subsequent TEVC (Figure 4A). Controls received the same treatment except that DA was omitted. Tetrodotoxin (TTX) was incorporated into these experiments because bath application of PKA antagonists caused cessation of a rhythmic network output (Figure 1B). Thus, to standardize both activity and drugs across experiments, (no Rp-cAMP, Rp-cAMP, 5 nM DA and 5 nM DA + Rp-cAMP) TTX was included in all treatment groups to block rhythmic network output. Previous experiments have demonstrated that co-application of TTX with DA did not affect the DA induced persistent increase in I_A_ G_max_[4]. Rp-cAMP blocked the DA induced persistent increase in I_A_ G_max_ (ANOVA, F_3, 22_ = 3.697, p = 0.027, Tukey’s post hoc, Rp-cAMP + DA vs TTX Ctrl, n.s., Rp-cAMP + DA vs TTX + DA, p < 0.05, Figure 4B).

### Erk activation is required for the DA mediated increase in I_A_ G_max_

**Figure 5:**
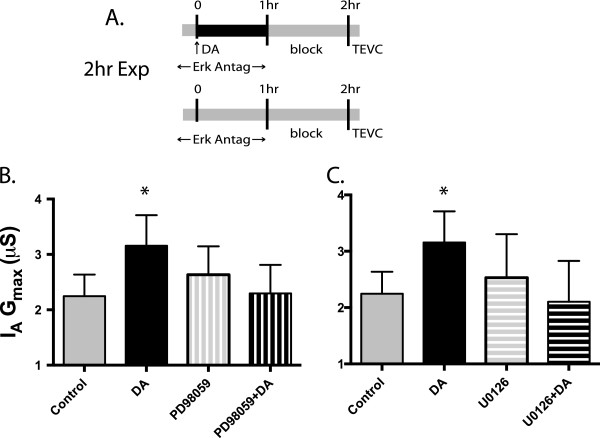
**Erk antagonists inhibit DA induced increase in I**_**A **_**G**_**max**_**. A**. Erk antagonists were applied with and without DA for 1 hr to an ongoing rhythm, followed by 1 hr of blocking saline and TEVC. Erk antagonists were applied 10 min prior to DA application. **B**. 50 μM PD98059 blocked the DA induced increase in I_A_ G_max_. **C**. 50 μM U0126 also blocked the DA induced increase in I_A_ G_max_. These data show that Erk is necessary to produce the persistent DA effect. Both data sets were analyzed by ANOVA, with Dunnet’s post hoc tests that compared each treatment group to control and plotted as mean ± S.D.

Erk has been shown to positively regulate mTOR activity through a number of mechanisms [28,37], and Erk signaling is necessary for mTOR mediated, forskolin (adenyl cyclase activator) induced, late-phase LTP [38]. However, depending upon the cell type, increased cAMP can activate [39] or inhibit [40] the Erk signaling pathway. To test whether Erk was involved in mediating the DA induced persistent increase in LP I_A_ G_max_ we used the indirect Erk antagonists PD98059 and U0126. Both drugs act on the mitogen-activated protein kinase kinases (MEK1/2) immediately upstream of Erk to prevent activation through phosphorylation. We co-applied either PD98059 or U0126 with or without 5 nM DA for 1 hr, followed by a 1 hr block and TEVC (Figure 5A). We compared the results of each drug to saline control and DA alone. Both drugs blocked the DA induced increase in I_A_: PD98059, Figure 5B, ANOVA F_3,20_ = 4.125, p = 0.019, Dunnet’s post hoc, ctrl vs DA, p < 0.05, ctrl vs PD98059, n.s., ctrl vs PD98059 + DA, n.s.. U0126, Figure 5C, ANOVA F3,19 = 3.133, p = 0.049, Dunnet’s post hoc ctrl vs DA, p < 0.05, ctrl vs U0126, n.s., ctrl vs U0126 + DA, n.s.. These data show that Erk activation is required for the persistent increase in I_A_ G_max_.

### U0126 affects the time constant of inactivation

**Figure 6:**
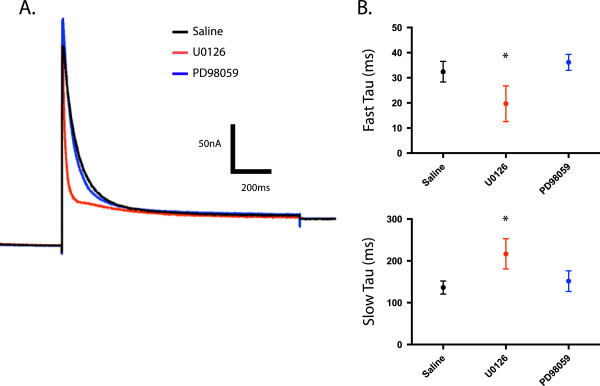
**U0126 alters the time constants of inactivation. A**. Representative two-electrode voltage clamp I_A_ recordings for Saline, U0126, PD98059 treatment groups. Overlaid traces are leak-subtracted currents elicited by a step to +20 mV after a -90 mV prepulse. **B**. Kinetics of I_A_ inactivation were determined by fitting the +20 mV current traces with a double exponential function. The mean fast (top panel) and slow (bottom panel) time constants (τ) of inactivation were plotted ± S.D for Saline, U0126, and PD98059 treatment groups. Data from drug alone and drug with 5 nM DA samples were pooled, as both means and variance between two groups were not different (n ≥ 10 for each group). Asterisks indicate significant differences from control (saline). Both the fast and slow τ values for U0126 were significantly different than PD98059 or Saline. Saline and PD98059 were not significantly different. Data were analyzed with ANOVAs and Tukey’s post hoc tests.

Shal (Kv4) channels mediate I_A_ in pyloric neurons [20-22]. Shal (Kv4) proteins are well conserved across species [41]. Previous work using U0126 has shown that it interacts directly with the rat Kv4.2 channel (a mammalian A-type K channel), causing an acceleration of inactivation of the channel [42]. To determine if U0126 had a similar effect on *Panulirus* A-type K channels, we determined the time constants of inactivation by fitting I_A_ inactivation with a double exponential function (Clampfit) (Figure 6A). We found that both fast and slow time constants were significantly different in the presence of U0126; the fast time constant was accelerated 40% by U0126 compared to saline, while the slow time constant was lengthened by 59%. PD98059, which also blocked the persistent effect of DA on LP I_A_, had no direct effect on A-channel inactivation kinetics (Fast τ, Figure 6B top panel, ANOVA F_2,28_ = 30.53, p < 0.0001, Tukey’s post hoc, U0126 vs Saline p < 0.0001, U0126 vs PD98059, p < 0.0001, PD98059 vs Saline, ns; Slow τ, Figure 6B bottom panel, ANOVA, F_2, 28_ = 25.65, p < 0.0001, Tukey’s post hoc, U0126 vs Saline p < 0.0001, U0126 vs PD98059, p < 0.0001, PD98059 vs Saline n.s., Figure 6B). This work supports the findings of Yuan *et al*., that the drug has an effect through direct interaction with the channel and that further, this effect may be present in many A-type K channels, given its presence in both mammals and crustaceans.

## Discussion

### Tonic DA regulates I_A_ in a time and dose dependent manner

We showed that dopaminergic tone influences I_A_ density. In the absence of tonic DA, average I_A_ G_max_ decreased by 13% over the course of 1 hr. Average I_A_ G_max_ did not decrease during a 1 hr application of ≥500 pM DA, but dropped to control levels when DA was removed. Average I_A_ G_max_ increased by ~10% during a 1 hr application of 5 nM DA and the increase was sustained for at least 5 hrs after removal of DA. 5 μM DA produced the same persistent increase in LP I_A_ G_max_[4]. We interpret the data to mean that dopaminergic tone acts at high affinity D1Rs to persistently augment LP I_A_ density. Our findings are consistent with previous work that suggests that tonic application of modulators can regulate surface expression of ion channels [43-45].

One interpretation of these data is that dopaminergic tone increases the ratio of the rate of shal channel insertion into versus removal from the plasma membrane. Upon removal of DA, the ratio will decrease, and I_A_ will decline according to the half-life of the channel. Since the DA-induced increase is translation-dependent, it is tempting to speculate that DA increases the pool of shal channels available for insertion. Alternatively, or in addition, it is also possible that DA might alter the subunit composition of the shal channels by incorporating different shal isoforms into the tetrameric channel [22] or by altering auxiliary subunits that interact with the alpha subunits [46], which are known to influence conductance [47]. It is also possible that DA alters levels of proteins involved in trafficking or endocytosis of shal channels. Since TEVC was always performed in the presence of TTX to block activity, we cannot rule out the possibility that decreases in activity may also contribute to changes in I_A_ G_max_. Indeed, both neuronal activity acting through changes in Ca^2+^ and neuromodulators can alter cAMP levels in arthropods via the adenylyl cyclase, rutabaga [48,49].

### Immediate and persistent regulation of I_A_ both utilize cAMP-PKA axis

The immediate and persistent effects of DA that decrease and increase I_A,_ respectively, are both mediated by a DA activated increase in cAMP and PKA activity [14]. It is unclear where the pathways diverge. LP cells express two different D1Rs: D1αPan and D1RβPan [7,14]. These distinct receptors could mediate the observed high and low affinity effects. This need not be the case. Receptors exist in multiprotein signaling complexes called signalplexes [50-52] and the same receptor could be incorporated into distinct signalplexes that generate unique cAMP signals. It has been demonstrated that agonists acting at receptors that positively couple with cAMP can simultaneously generate large, temporally complex, local signals and sustained global signals [53-56]. Compartmentilization of cAMP signaling has been demonstrated to be critical in mediating differential downstream effects of cAMP and preventing non-specific activity of cAMP effectors [30]. cAMP signals can be constrained by differential PKA compartmentalization via A Kinase anchoring proteins (AKAPs) [31] and/or by differential phosphodiesterase localization [54]. D1Rs are predominately localized to terminals in fine neurites [14]. Previous cAMP imaging studies on STG neurons showed that continuous application of modulators, including DA, initially produced a cAMP signal in the terminals that eventually spread throughout the cell [57]. Since the persistent effect is induced by continuous exposure to DA, that could result in more global changes in shal channels than the immediate effect.

### PKA and ERK contribute to the persistent increase in LP I_A_ G_max_

Erk activation is required for the persistent increase in I_A_ G_max_. Both MEK antagonists blocked the persistent effect when co-applied with 5 nM DA. It is not clear if ERK and PKA are acting in parallel or series. The intracellular signaling pathway mediating the persistent increase in LP I_A_ shows a remarkable overlap with many proteins involved in L-3, 4-dihydroxyphenylalanine (L-DOPA) induced dyskinesia (LID) [58-60]. Specifically, both pathways involve a D1R mediated increase in cAMP, PKA activation, increase in Erk activity, and finally mTORC1 activation. LID is attenuated by PKA [61] and mTOR antagonism [62]. Independent dual activation of cAMP/PKA axis and Erk by D1Rs has been observed in LID, where L-DOPA treated Gα_olf_ deficient mice showed decreased PKA phosphorylation, but no change in Erk activation [63]. The Erk pathway has multiple points of interaction with proteins affecting mTOR activity [28], and based on this data, it is impossible to say which protein pathways mediate this effect. Interestingly, the neurotrophic factor Neuritin, which also increases I_A_ (Kv4.2) in a dose and time dependent manner in mammalian neurons, requires both Erk and mTOR [64], suggesting many components of modulatory tone may act together to determine I_A_ density.

## Conclusions

DA acts at high affinity receptors to increase I_A_ G_max_ through a translation dependent mechanism that requires a functional D1R-PKA axis, Erk and mTOR.

## Methods

### Animals

California spiny lobsters, *Panulirus interruptus,* were purchased from Catalina Offshore Products (San Diego, CA) and Marinus Scientific (Long Beach, CA) and housed in saltwater aquaria at Georgia State University (Atlanta, GA). Animals were a mix of both male and females.

This research was carried out in accordance with the IACUC standards for use of animals in research at Georgia State University.

### Pharmacology

All drugs were administered to the STG via superfusion. DA was administered for 1 hour in all cases. To minimize oxidation, DA was made fresh and exchanged after 30 min. Dosages of PKA antagonist Rp-cAMP (1 mM) (Sigma), and mTOR antagonist rapamycin (100 nM) (Sigma) were chosen based previously established effective doses in the STG [4,14]. The ePAC agonist (Tocris) was applied at 50 μM [36]. ErK activity was blocked by the use of MEK antagonists PD98059 (50 μM, Invivogen) and U0126 (50 μM, Tocris) based on previously shown effective dosages in the white shrimp, *F. indicus*[65], and dissolved in DMSO. Drugs were applied to the preparation 10 min before the application of DA.

### STNS Dissection, Pyloric cell identification

Lobsters were anaesthetized on ice for at least 30 min, followed by the dissection of the STNS, as previously described [66]. The STNS was pinned in a Sylgard-lined dish. The STG was desheathed and petroleum jelly well was constructed around it. Using a Dynamax peristaltic pump (Rainin), the STG was superfused with *Panulirus* (*P*.) saline (in mM: 479 NaCl, 12.8 KCl, 13.7 CaCl_2_, 39 Na_2_SO_4_, 10 MgSO_4_, 2 Glucose, 4.99 HEPES, 5 TES; pH 7.4).

Experiments were performed at room temperature. Temperature was continuously monitored with a miniature probe in the bath. The temperature changed by less than 1°C throughout the course of the day (the change ranged from 0.1 to 0.9°C on any given day), and by only 3°C across all experiments (19-22°C).

Cells were identified using previously described standard intracellular and extracellular recording techniques. Intracellular somatic recordings (such as those seen in Figure 1B) were obtained using 20–40 MΩ glass microelectrodes filled with 3 M KCl and Axoclamp 2B or 900A amplifiers (Molecular Devices, Foster City, CA). Extracellular recordings of identified motor neurons were obtained using a differential AC amplifier (A-M Systems, Everett, WA) with stainless steel pin electrodes. LP neurons were identified by their distinct waveforms, the timing of their voltage oscillations, and correlation of spikes on the extracellular and intracellular recordings (Figure 1B).

### Two-electrode voltage clamp

A portion of the stomatogastric nerve was isolated in a petroleum jelly well containing isotonic sucrose; descending inputs were removed by cutting the STN in the sucrose bath 1 hour prior to TEVC. The STG was superfused continuously with blocking saline, which consisted of *P.* saline containing picrotoxin (10^-6^ M) to block glutamatergic synaptic inputs and voltage-dependent ion channel blockers: tetrodotoxin (TTX, 100 nM, I_Na_), tetraethylammonium (TEA, 20 mM, I_K(V)_ and I_K(Ca)_), and cadmium chloride (CdCl_2_, 200 μM, I_Ca_). LP cells were impaled with two low resistance microelectrodes (8–10 MΩ) filled with 3 M KCl. The holding potential was -50 mV. I_A_ activation was measured by two different protocols, A and B. Protocol A: I_A_ was elicited by a series of depolarizing steps (500 ms) ranging from -50 to +60 mV in 10 mV increments that were or were not preceded by a 200 ms prepulse to -90 mV to remove resting inactivation of A type K + channels. I_A_ was obtained by digitally subtracting the current obtained without a prepulse from currents obtained with a prepulse. After digital subtraction, the peak current was converted to conductance (G = I_peak_/(V_m_-E_k_), plotted against voltage and fit using a 1^st^ order Boltzmann equation to determine the voltage of half activation and maximal conductance. Protocol B: here the voltage protocol was modified to minimize the effects of repeated depolarization. This protocol was only used in the experiments shown in Figure 2D. I_A_ activation was measured with 8 depolarizing steps that ranged from -50 mV to +20 mV, and the minimum tail current was subtracted from peak current for each sweep. Data was again fit with a 1^st^ order Boltzmann equation to determine the voltage of half activation and maximal conductance. Steady state inactivation was measured by a series of sweeps that varied the range of the 200 ms prepulse from -110 to -20 mv in 10 mV increments followed by a constant step to 20 mV (500 ms). To further isolate I_A,_ a depolarizing prepulse to -20 mV, followed by a test pulse to 20 mV was digitally subtracted from each inactivation trace. Peak current was plotted for each voltage and fit with a 1^st^ order boltzmann equation to derive voltage of half inactivation.

### Statistical analysis

The data were checked for normality and analyzed using parametric statistics. Data were analyzed using Prism Statistical software package (Graphpad) and SAS version 8.1 (SAS Institute Inc.). Significance threshold was set at p < 0.05 in all cases. Statistical outliers were excluded based on Chauvenet’s Criterion. Means are presented ± Standard Deviation.

## Competing interests

The authors declare that they have no competing interests.

## Authors’ contributions

ER designed and carried out experiments, performed statistical analysis, and drafted the manuscript. WK performed experiments and analyzed data. XJ performed experiments, aided in the experimental design and data analysis. LL provided material support, aided in the design of experiments, and data interpretation. DB conceived of the study, participated in its design and coordination, and wrote and edited significant portions of the MS. All authors read and approved the final manuscript.
